# Molecular mechanisms of renal and extrarenal manifestations caused by inactivation of the electrogenic Na^+^-HCO_3_^−^ cotransporter NBCe1

**DOI:** 10.3389/fphys.2013.00270

**Published:** 2013-10-01

**Authors:** George Seki, Shoko Horita, Masashi Suzuki, Osamu Yamazaki, Tomohiko Usui, Motonobu Nakamura, Hideomi Yamada

**Affiliations:** ^1^Department of Internal Medicine, School of Medicine, The University of TokyoTokyo, Japan; ^2^Department of Ophthalmology, School of Medicine, The University of TokyoTokyo, Japan

**Keywords:** NBCe1, pRTA, short stature, ocular abnormalities, migraine, enamel abnormalities, dominant negative effect

## Abstract

The electrogenic Na^+^-HCO_3_^−^ cotransporter NBCe1 plays an essential role in bicarbonate absorption from renal proximal tubules, but also mediates the other biological processes in extrarenal tissues such as bicarbonate secretion from pancreatic ducts, maintenance of tissue homeostasis in eye, enamel maturation in teeth, or local pH regulation in synapses. Homozygous mutation in NBCe1 cause proximal renal tubular acidosis (pRTA) associated with extrarenal manifestations such as short stature, ocular abnormalities, enamel abnormalities, and migraine. Functional analyses of NBCe1 mutants using different expression systems suggest that at least a 50% reduction of the transport activity may be required to induce severe pRTA. In addition to functional impairments, some NBCe1 mutants show trafficking defects. Some of the pRTA-related NBCe1 mutants showing the cytoplasmic retention have been shown to exert a dominant negative effect through hetero-oligomer complexes with wild-type NBCe1 that may explain the occurrence of extrarenal manifestations in the heterozygous carries of NBCe1 mutations. Both NBCe1 knockout (KO) and W516X knockin (KI) mice showed very severe pRTA and reproduced most of the clinical manifestations observed in human pRTA patients. Functional analysis on isolated renal proximal tubules from W516X KI mice directly confirmed the indispensable role of NBCe1 in bicarbonate absorption from this nephron segment. In this review, we will focus on the molecular mechanisms underling the renal and extrarenal manifestations caused by NBCe1 inactivation.

## Introduction

The SLC4 bicarbonate transport family mediates important roles in the regulation of extracellular and intracellular pH (Romero et al., 2013). Among them, the electrogenic Na^+^-HCO_3_^−^ cotransporter NBCe1 encoded by the *SLC4A4* gene plays an essential role in bicarbonate absorption from renal proximal tubules (Romero and Boron, 1999). Consistent with this view, loss-of-function mutations in NBCe1 result in severe proximal renal tubular acidosis (pRTA), with the blood bicarbonate concentration often less than 10 mM (Igarashi et al., 1999). In extrarenal tissues, NBCe1 plays other biological functions such as the maintenance of corneal transparency, pancreatic-duct bicarbonate secretion, enamel maturation in teeth, and local pH regulation in neuronal synapses. Indeed, homozygous mutations in NBCe1 are associated with the ocular and other extrarenal manifestations. This review will focus on the molecular mechanisms of renal and extrarenal manifestations caused by NBCe1 inactivation.

## Molecular identification of NBCe1

In 1983, Boron and Boulpaep clarified the mechanism of HCO_3_^−^ transport in the basolateral membrane of isolated salamander renal proximal tubules. They identified the existence of electrogenic Na^+^-dependent HCO_3_^−^ transport activity now known as NBCe1 (Boron and Boulpaep, 1983). They speculated that the decrease in cell pH due to the operation of basolateral NBCe1 is responsible for the activation of luminal Na^+^/H^+^ exchanger NHE3, which is necessary for the net bicarbonate absorption process.

Similar Na^+^-coupled HCO_3_^−^ transport activities have been found in other tissues such as corneal endothelium (Jentsch et al., 1984), glial cells (Deitmer and Schlue, 1987), heart (Dart and Vaughan-Jones, 1992), colon (Rajendran et al., 1991), and pancreatic duct cells (Ishiguro et al., 1996a, b). Romero and colleagues have established the molecular identity of these transport activities by the first successful cloning of NBCe1 (Romero et al., 1997). They succeeded in cloning salamander renal NBCe1 by searching for the activity of an electrogenic Na^+^-coupled HCO_3_^−^ transporter heterologously expressed in *Xenopus* oocytes.

It is now known that NBCe1 has three major variants. Among them, the renal variant NBCe1-A, transcribed from an alternate promoter in intron 3 (Abuladze et al., 2000), is predominantly expressed in the basolateral membrane of renal proximal tubules, where it represents a majority of the bicarbonate exit pathway (Boron, 2006). NBCe1-B, first cloned from pancreas (Abuladze et al., 1998) and transcribed from the dominant promoter in exon 1 (Abuladze et al., 2000), is widely expressed in many tissues. NBCe1-B is thought to mediate several distinct biological processes such as pancreatic-duct bicarbonate secretion (Ishiguro et al., 1996a, b), the maintenance of corneal transparency (Bok et al., 2001; Usui et al., 2001), and the regulation of synaptic pH (Chesler, 2003; Suzuki et al., 2010). NBCe1-A and NBCe1-B differ only at the N-terminus, where the first 41 amino acids of NBCe1-A replace the first 85 amino acids of NBCe1-B (Abuladze et al., 2000). On the other hand, NBCe1-C, predominantly expressed in the brain (Bevensee et al., 2000), is identical to NBCe1-B except for the C-terminus, where the last 61 amino acids of NBCe1-C replace the last 46 amino acids of NBCe1-B. The physiological significance of NBCe1-C remains to be established. NBCe1-D and NBCe1-E, which lack 9 amino acids in exon 6 of NBCe1-A and NBCe1-B, respectively, have been identified from mouse reproductive tract tissues (Liu et al., 2011).

## Functional differences of NBCe1 variants

One of the major functional differences between NBCe1-A and NBCe1-B is the transport direction and transport stoichiometry. Thus, NBCe1A in the basolateral membrane of renal proximal tubules mediates bicarbonate efflux from cells most likely with a 1Na^+^ to 3HCO_3_^−^ stoichiometry (Yoshitomi et al., 1985). On the other hand, NBCe1-B in the basolateral membrane of pancreatic ducts mediates bicarbonate influx into cells presumably with a 1Na^+^ to 2HCO_3_^−^ stoichiometry (Gross et al., 2001a; Steward et al., 2005). However, these different stoichiometric ratios may not originate from the intrinsic properties of NBCe1 variants, but rather may be due to cellular environments and/or tissue specific factors. For example, NBCe1-B, when transfected into renal proximal tubular cells, can function with a 1Na^+^ to 3HCO_3_^−^ stoichiometry (Gross et al., 2001b). Furthermore, NBCe1-A in isolated renal proximal tubules can operate with either a 1Na^+^ to 2HCO_3_^−^ stoichiometry or a 1Na^+^ to 3HCO_3_^−^ stoichiometry, depending on the incubation conditions (Seki et al., 1993; Muller-Berger et al., 1997). NBCe1A expressed in *Xenopus* oocytes can also change transport stoichiometry depending on the cell Ca^2+^ concentrations (Muller-Berger et al., 2001). Interestingly, even if NBCe1-A operates with a 1Na^+^ to 2HCO_3_^−^ stoichiometry, it may still function as the basolateral bicarbonate exit pathway in renal proximal tubules (Boron and Boulpaep, 1983; Seki et al., 1993, 1996).

Another functional difference among NBCe1 variants is the presence of an autoinhibitory domain in the N terminus of NBCe1-B and NBCe1-C. Thus, the activities of NBCe1-B/C expressed in *Xenopus* oocytes correspond to only 20–30% of the activity of NBCe1-A (McAlear et al., 2006). Deletion of the specific N-terminal region of NBCe1-B/C significantly enhanced the NBCe1 activity, consistent with the relief of autoinhibitory action of this N-terminal region. Subsequently, an inositol 1,4,5-trisphosphate receptor (IP_3_R) binding protein termed IRBIT was found to specifically bind to the N-terminal region containing the autoinhibitory domain and activate NBCe1-B/C (Shirakabe et al., 2006; Yang et al., 2011b). IRBIT, originally identified by its ability to bind to IP_3_R, is dissociated from IP_3_R in the presence of physiological concentrations of IP_3_, and this process is thought to be important for the regulation of IP_3_R activity (Ando et al., 2003, 2006). In *Xenopus* oocytes, coexpression of IRBIT can markedly activate NBCe1-B without changing its membrane expression (Shirakabe et al., 2006). On the other hand, IRBIT does not activate NBCe1-A, which is lacking the autoinhibitory domain. These results strongly suggest that the binding of IRBIT to the NBCe1-B/C-specific N-terminal region may somehow induce conformational changes, thereby relieving its autoinhibitory action.

Subsequent studies have clarified that IRBIT has a central role in fluid and bicarbonate secretion by activating both NBCe1-B and the cystic fibrosis transmembrane conductance regulator CFTR in secretory epithelia such as pancreatic ducts (Yang et al., 2009). In addition to the direct stimulatory effect, IRBIT may stimulate NBCe1-B and CFTR indirectly by recruiting protein phophatase-1, reversing the inhibitory action of the with-no-lysine kinases (WNK)/Ste20-related proline/alanine-rich kinase (SPAK) pathway (Yang et al., 2011a). Interestingly, a positively charged module within the amino acids 37–62 of NBCe1-B may be responsible for the activation by IRBIT, as well as the inactivation by WNKs/SPAK. Furthermore, a similar domain is found in the other bicarbonate transporters such as the electroneutral Na^+^-HCO_3_^−^ cotransporter NBCn1-A, the Na^+^-dependent Cl^−^/HCO_3_^−^ exchanger NDCBE-A, or the Cl^−^/HCO_3_^−^ exchanger SLC26a6, as well as in the Cl^−^ channel CFTR. IRBIT and WNKs/SPAK may regulate all these transporters through similar mechanisms depending on this domain (Hong et al., 2013). This domain may be also responsible for the PIP_2_-mediated activation of NBCe1-B/C (Thornell et al., 2012). On the other hand, IRBIT may mediate the synergic activation of CFTR and SLC26a6 by Ca^2+^ and cAMP through mechanisms involving the dissociation from IP_3_R, and the translocation of CFTR and SLC26a6 to the plasma membrane (Park et al., 2013).

## Renal phenotypes caused by NBCe1 mutations

Renal proximal tubules reabsorb more than 80% of the filtered bicarbonate, and an impairment of this process results in pRTA. pRTA usually occurs as one manifestation of generalized defects in proximal tubular functions known as Fanconi syndrome. An isolated defect in proximal bicarbonate absorption is rare, and mostly transient in infants or children. Hereditary isolated pRTA is extremely rare and divided into two types by the following clinical features; (1) stunted growth without other predominant clinical features, and (2) stunted growth with ocular abnormalities such as band keratopathy, cataracts, and glaucoma (Rodriguez Soriano et al., 1967; Nash et al., 1972; Igarashi et al., 1994).

**Figure 1:**
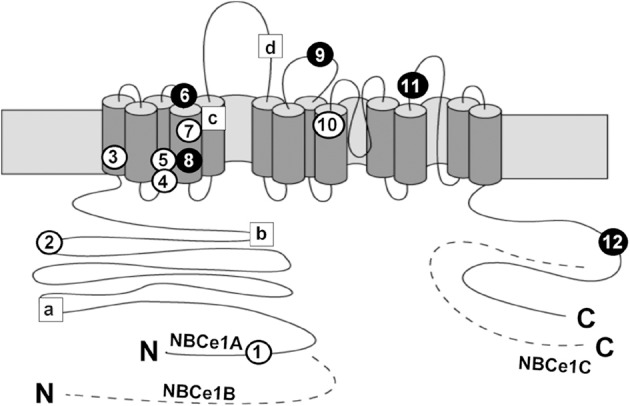
**Topological model and mutations of NBCe1.** Circles show the localization of the following pRTA-related mutations: Q29X (1), R298S (2), S427L (3), T485S (4), G486R (5), R510H (6), W516X (7), L522P (8), 2311 delA (9), A799V (10), R881C (11), and S982NfsX4 (12), where black circles indicate the association with migraine. Squares show the localization of the following SNPs: E122G (a), S356Y (b), K558R (c), and N640I (d). The dashed segments represent the specific regions of NBCe1-B or NBCe1-C; N and C denote the N- and C-termini, respectively.

In 1999, Igarashi and colleagues identified homozygous NBCe1 mutations in two pRTA patients with short stature and ocular abnormalities (Igarashi et al., 1999). Until now, 12 NBCe1 mutations have been identified in pRTA patients with similar ocular abnormalities. As shown in Figure 1, these mutations consist of eight missense mutations (R298S, S427L, T485S, G486R, R510H, L522P, A799V, and R881C), two non-sense mutations (Q29X and W516X), and two frame shift mutations (N721TfsX29 and S982NfsX4) (Igarashi et al., 1999, 2001; Dinour et al., 2004; Inatomi et al., 2004; Horita et al., 2005; Demirci et al., 2006; Suzuki et al., 2008, 2010; Lo et al., 2011). Among them, Q29X, which lies in the NBCe1-A-specific N-terminal region, is expected to diminish NBCe1-A activity completely, but leave NBCe1-B/C function intact (Igarashi et al., 2001). On the other hand, the C-terminal mutant S982NfsX4 is expected to introduce a frameshift in exon 23 and a premature stop codon for NBCe1-A/B, but abolish the translation of NBCe1-C, the C-terminal variant lacking exon 24 (Suzuki et al., 2010). All the other mutations lie in the common region of NBCe1 variants.

Consistent with the major role of NBCe1-A in bicarbonate absorption from renal proximal tubules, most of these pRTA patients presented with a severe metabolic acidosis evidenced by a blood bicarbonate concentration often less than 10 mM. The resultant severe acidemia seems to be responsible for short stature found in most of these pRTA patients, because alkali therapy, if started early in the life and successfully continued for a sufficient period, is able to at least partially improve the growth rate (Shiohara et al., 2000). Furthermore, the two sisters with a S982NfsX mutation had a relatively mild acidosis with a blood bicarbonate concentration of around 15 mM, but had normal stature (Suzuki et al., 2010).

Two types of NBCe1-deficient mice, NBCe1 knockout (KO) and W516X knockin (KI) mice have been produced. They showed very severe acidemia with a blood bicarbonate concentration around 5 mM (Gawenis et al., 2007; Lo et al., 2011). Both mice also showed growth retardation, hyperaldosteronism, anemia, splenomegaly, and early death before weaning. Alkali therapy significantly prolonged survival and partially improved growth retardation and bone abnormalities in the W516X KI mice (Lo et al., 2011). Functional analysis using isolated renal proximal tubules obtained from the W516X KI mice confirmed the indispensable role of NBCe1 in bicarbonate absorption from this nephron segment (Lo et al., 2011). The severe phenotypes observed in NBCe1-deficient mice suggest that the compensatory mechanism for dysfunctional renal proximal bicarbonate absorption is less efficient in mice than in humans. Consistent with this view, a mild metabolic acidosis has been found in both of the heterozygous NBCe1-deficient mice (Gawenis et al., 2007; Lo et al., 2011), but not in the heterozygous human carriers of the pathological NBCe1 mutations.

In contrast to these NBCe1 deficient mice, NHE3 KO mice showed only a mild metabolic acidosis with a bicarbonate concentration around 20 mM (Schultheis et al., 1998). These mice had significant residual NHE activity in the apical membrane of renal proximal tubules that might represent NHE8 activity (Choi et al., 2000; Goyal et al., 2003). Functional analysis using isolated renal proximal tubules from NHE3/8 double-KO mice confirmed this view (Baum et al., 2012). Surprisingly, however, the acidosis of NHE3/8 double-KO mice was also mild and comparable to that in NHE3 KO mice (Baum et al., 2012).

## Mechanisms of NBCe1 inactivation

Functional analyses using different expression systems suggest that at least a 50% reduction in NBCe1 activity is required to induce severe pRTA (Igarashi et al., 1999; Horita et al., 2005; Suzuki et al., 2008). However, no tight relationship exists between the degree of NBCe1-A inactivation and the severity of acidemia (Horita et al., 2005; Suzuki et al., 2008, 2010). In addition to the pure functional impairments, trafficking defects may be also involved in the pathogenesis of renal and extrarenal manifestations caused by NBCe1 mutations. As discussed below, cytosolic retention of NBCe1 mutations may be associated with migraine (Suzuki et al., 2010). NBCe1 can form oligomer complexes (Kao et al., 2008), and NBCe1 mutants showing cytosolic retention may have a dominant-negative effect in common (Yamazaki et al., 2013).

Topological analysis based on the substituted cysteine accessibility method suggests that most NBCe1 missense mutations reside in positions buried deep in the protein complex/lipid bilayer that may be important for either protein folding, helices packing, or ion translocation (Zhu et al., 2010). Among these mutations, T485S is of special interest because the corresponding codon in the electroneutral anion exchangers (AEs1-3) is serine (Romero and Boron, 1999). T485S reaches the basolateral membrane in MDCK cells and retains 30–50% of wild-type NBCe1 activity in ECV304 or HEK293 cells (Horita et al., 2005; Suzuki et al., 2008, 2010). Recently, Zhu and colleagues reported an intriguing possibility that the T485S mutant may actually work as an electroneutral Na^+^-HCO_3_^−^ cotransporter in HEK293 cells (Zhu et al., 2013). Although the analysis by cell-pH measurements reveals that the T485S has ~50% of wild-type transport function, the analysis by patch-clamp fails to detect electrogenic activity of T485S. The authors speculate that the electroneutral T485S mutant may transport Na^+^-HCO_3_^−^ in the reverse direction from blood into tubular cells, representing a novel pathogenic mechanism for pRTA (Zhu et al., 2013). Unfortunately, the absence of T485S surface expression precludes the precise analysis of its electrogenicity in *Xenopus* oocytes (Horita et al., 2005). On the other hand, Chen and Boron suggest that the predicted fourth extracellular loop corresponding to amino acids 704–735 may have an important role in the electrogenicity of NBCe1 (Chen et al., 2011).

Arg^298^ in the C-terminal cytoplasmic domain of NBCe1-A was also predicted to reside in a solvent-inaccessible location, which may be associated with Glu^91^ or Glu^295^ via H-bonding and charge-charge interactions (Chang et al., 2008). This continuous chain of interconnected polar residues may be important for the HCO_3_^−^ transporting ability of NBCe1.

In addition to pRTA-related mutations, non-synonymous single nucleotide polymorphisms (SNPs) in NBCe1 have also been analyzed as shown in Figure 1. Among 4 SNPs (E122G, S356Y, K558R, and N640I), only the K558R variant, which is predicted to lie in transmembrane segment 5, is found to reduce the NBCe1-A activity significantly without changing the trafficking behavior or the apparent extracellular Na^+^ affinity (Yamazaki et al., 2011). At present, the clinical significance of K558R SNP remains unknown.

## Ocular phenotypes caused by NBCe1 mutations

pRTA patients with homozygous NBCe1 mutations invariably presented with ocular abnormalities, typically consisting of band keratopathy, glaucoma, and cataract, indicating that the normal transport activity of NBCe1 in eye is indispensable for the maintenance of tissue homeostasis (Suzuki et al., 2012).

**Figure 2:**
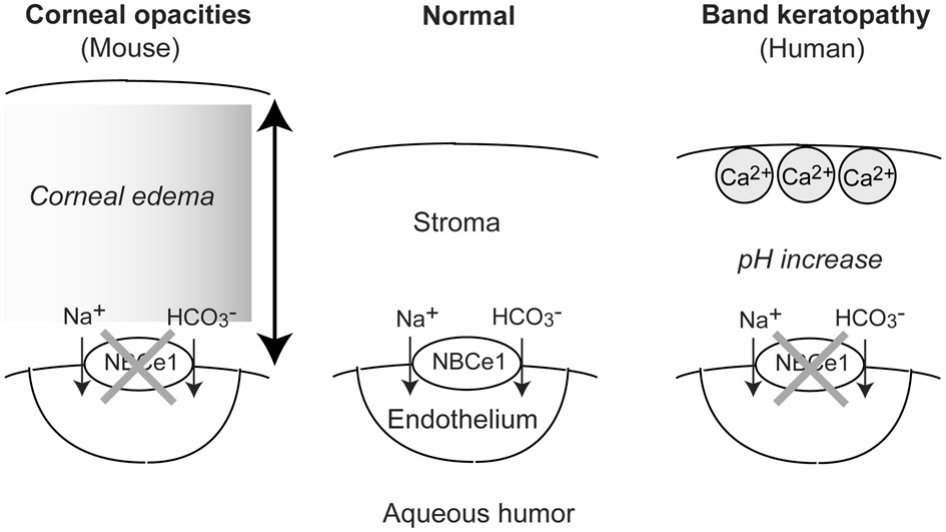
**Roles of NBCe1 in the corneal endothelium.** In mouse, NBCe1 inactivation may induce corneal edema, resulting in corneal opacities. In human, NBCe1 inactivation may increase local pH, resulting in band keratopathy.

In particular, the physiological role of NBCe1 has been established in the corneal endothelium. The electrogenic transport of sodium and bicarbonate into the aqueous humor by the corneal endothelium has been considered to be essential for corneal hydration and transparency (Hodson and Miller, 1976). The involvement of NBCe1 in this process has been suggested by the identification of electrogenic Na^+^-HCO_3_^−^ cotransport activity in cultured bovine corneal endothelial cells (Jentsch et al., 1984). Subsequently, Usui and colleagues obtained functional and molecular evidence for NBCe1 in cultured human corneal endothelial cells (Usui et al., 1999). Furthermore, immunohistological analysis confirmed the expression of NBCe1 in rat, human, and bovine corneal endothelium (Sun et al., 2000; Bok et al., 2001; Usui et al., 2001). Probably, NBCe1 inactivation impairs bicarbonate efflux by the corneal endothelium that may in turn increase the local pH within the corneal stroma. This increase in the local pH may facilitate local Ca^2+^ deposition causing band keratopathy (Usui et al., 2001). Notably, NBCe1 W516X KI mice, who survived more than 1 month with alkali therapy, showed corneal opacities due to corneal edema, indicating that NBCe1 plays an indispensable role in the maintenance of corneal transparency in mice (see Figure 2) (Lo et al., 2011).

NBCe1 is expressed in trabecular meshwork cells as well as in retina (Bok et al., 2001; Usui et al., 2001), both of which may be involved in the pathogenesis of glaucoma due to NBCe1 inactivation. The electrogenic Na^+^-HCO_3_^−^ cotransport activity compatible with NBCe1 was reported in human trabecular meshwork cells (Lepple-Wienhues et al., 1994). It is well-known that the trabecular meshwork is the main site regulating aqueous outflow in the human eye (Bill, 1975). Accordingly, it is tempting to speculate that the inactivation of NBCe1 in trabecular meshwork cells may be responsible for the occurrence of high-tension glaucoma usually observed in the pRTA patients with homozygous NBCe1 mutations (Suzuki et al., 2010, 2012). Interestingly, the pRTA patient carrying the homozygous Q29X mutation also presented with bilateral high-tension glaucoma (Igarashi et al., 2001). These results suggest that although NBCe1B may be the predominant variant in the ocular tissues, NBCe1-A may also play a significant role in eye (Bok et al., 2001; Usui et al., 2001).

On the other hand, some of the family members carrying the heterozygous NBCe1 S982NfsX4 mutation were found to have normal-tension glaucoma without pRTA (Suzuki et al., 2010). The S982NfsX4 mutant was predominantly retained in the endoplasmic reticulum (ER) in mammalian cells. When coexpressed with wild-type NBCe1, this mutant exerted a dominant-negative effect by forming hetero-oligomer complexes with wild-type NBCe1 (Suzuki et al., 2010). Because NBCe1 in retinal Müller cells may protect against excessive synaptic activity by counteracting the light-induced extracellular alkalosis (Borgula et al., 1989; Newman, 1999), the dominant-negative effect of S982NfsX4 in retina may be responsible for normal-tension glaucoma found in the heterozygous family members. We recently found that the cytosolic mutant L522P also had a similar dominant-negative effect (Yamazaki et al., 2013). However, we have no information about the effects of the heterozygous L522P mutation on human health because the pRTA patient carrying the homozygous L522P mutation was adopted at infancy (Demirci et al., 2006).

The lens is an avascular tissue, and ion transport by lens epithelium may be critical for the maintenance of lens homeostasis and integrity. Immunohistological analysis indeed detected the expression of NBCe1 in rat and human lens epithelium (Bok et al., 2001; Usui et al., 2001). Furthermore, electrogenic Na^+^-HCO_3_^−^ cotransport activity consistent with NBCe1 has been identified in cultured human lens epithelial cells (Usui et al., 2001). These results suggest that the inactivation of NBCe1 in lens epithelium may somehow impair lens homeostasis, resulting in cataract formation.

## Migraine and NBCe1 mutations

Migraine is a common, disabling, and multifactorial disorder, which affects more than 10% of the general population (Lipton et al., 2002). While a genetic factor certainly plays a substantial role in ordinary migraine, the precise genetic basis has been established only in familial hemiplegic migraine (FHM), a rare autosomal dominant subtype of migraine with aura. Surprisingly, the following three genes identified as the genetic basis for FHM all encode ion transporters: *CACNA1A* encoding the α1 subunit of voltage-gated neuronal Cav2.1 calcium channels (Ophoff et al., 1996), *ATP1A2* encoding the α2 subunit of Na^+^/K^+^ ATPase (De Fusco et al., 2003), and *SCN1A* encoding the neuronal voltage-gated sodium channel Nav1.1 (Dichgans et al., 2005). These mutations in FHM genes may cause migraine by enhancing neuronal excitability (Goadsby, 2007). Enhanced neurotransmitter release by *CACNA1A* mutations, excessive neuronal firing by *SCN1A* mutations, or impaired clearance of K^+^ and/or glutamate by *ATP1A2* mutations can all induce cortical spreading depression (CSD), which may be the underlying pathophysiological mechanism of migraine aura (Goadsby, 2007). NBCe1B in astrocytes may also regulate neuronal excitability by bicarbonate uptake upon depolarization, known as a depolarization-induced alkalinization DIA (Chesler, 2003). The net extracellular acidosis due to DIA may reduce neuronal excitability, especially because extracellular acidosis is known to inhibit excitatory NMDA receptors (Chesler, 2003).

**Figure 3:**
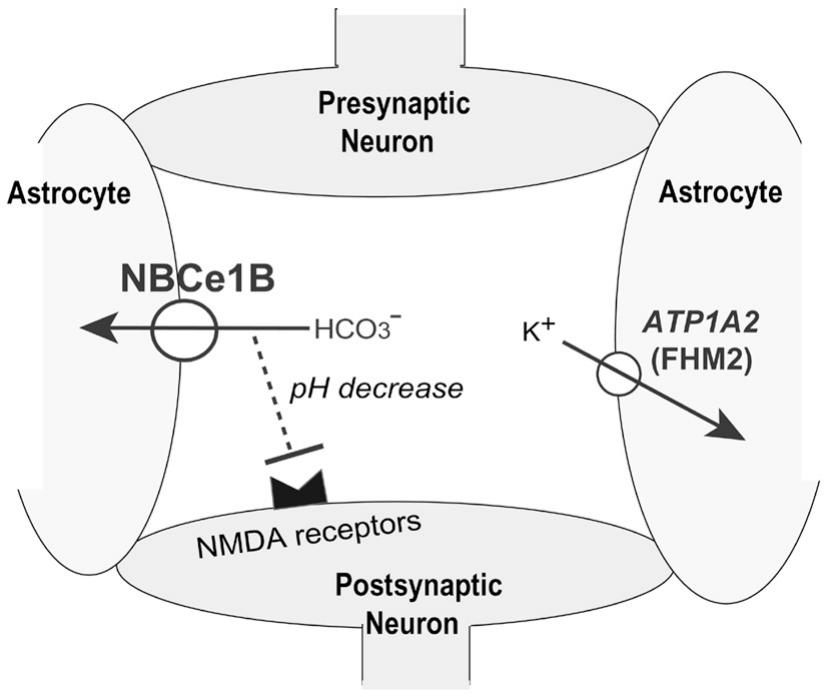
**Role of NBCe1B in synaptic pH regulation.** K^+^ uptake into astrocytes by *ATP1A2* may suppress neuronal excitation. NBCe1-B- mediated local pH decreases may also suppress neuronal excitation by inhibiting pH-sensitive NMDA receptors.

Consistent with a significant role of NBCe1 in the regulation of neuronal excitability, we identified two sisters with relatively mild pRTA, severe ocular abnormalities, and hemiplegic migraine. Genetic analysis excluded pathological mutations in *CACNA1A*, *ATP1A2*, and *SCN1A*, but identified the homozygous S982NfsX4 mutation in the C-terminus of NBCe1. As already mentioned, this mutant showed predominant ER retention in mammalian cells. Furthermore, we found that 4 additional patients carrying homozygous NBCe1 mutations had the following types of migraine: hemiplegic migraine with episodic ataxia in the patient with L522P (Demirci et al., 2006), migraine with aura in the patient with N721TfsX29 (Inatomi et al., 2004), and migraine without aura in the patients with R510H and R881C (Igarashi et al., 1999; Horita et al., 2005). All these mutations result in the lack of plasma membrane expression of NBCe1-B in C6 glioma cells. We therefore concluded that almost complete loss of NBCe1-B activity in astrocytes might cause migraine potentially through dysregulation of synaptic pH as shown in Figure 3 (Suzuki et al., 2010). We think that migraine due to NBCe1 mutations may be a primary headache most likely caused by dysfunctional local pH regulation in the brain.

## Enamel deficiency and NBCe1 mutations

During enamel maturation and crystal nuleation, large amounts of proton are released. The resultant decreases in the local pH to a very acidic range may inhibit further mineralization (Simmer and Fincham, 1995). Thus, homeostatic regulation of local pH is critical for normal enamel maturation. The involvement of NBCe1 in this process is suggested by the expression of this transporter in the basolateral membrane of ameloblasts (Paine et al., 2008).

Consistent with this view, the defect in enamel maturation is found in pRTA patients with NBCe1 mutations (Dinour et al., 2004; Inatomi et al., 2004). Furthermore, the enamel of NBCe1 KO mice was extremely hypomineralized and weak with an abnormal prismatic architecture (Lacruz et al., 2010). In ameloblast cells, NBCe1 mediates bicarbonate uptake from the basolateral membrane. On the other hand, the Cl^−^/HCO_3_^−^ exchanger AE2 may mediate bicarbonate secretion from the apical membrane (Lacruz et al., 2012). Local pH regulation through bicarbonate transport by NBCe1 and AE2 may be indispensable for normal enamel mineralization.

## Conclusion

As described in this review, NBCe1 plays diverse biological roles in many tissues. In particular, the essential role of NBCe1 in renal proximal bicarbonate absorption is evidenced by the severe pRTA caused by NBCe1 inactivation in both humans and mice. The clinical manifestations associated with NBCe1 inactivation uncover the other biological roles of NBCe1 in the extrarenal tissues. NBCe1 may also play an important role in pancreatic bicarbonate secretion. However, clinically evident pancreatitis has not been reported in pRTA patients with NBCe1 mutations, suggesting that other acid/base transporters in the basolateral membrane of pancreatic ducts may be able to at least partially compensate for the loss of NBCe1 activity (Steward et al., 2005). On the other hand, pathological mutations in NHE3 have not been found in human pRTA patients. Furthermore, a puzzling question remains unanswered as to why NHE3/8 double-KO mice with the minimal residual NHE activity in the apical membrane of renal proximal tubules present with only a mild metabolic acidosis.

### Conflict of interest statement

The authors declare that the research was conducted in the absence of any commercial or financial relationships that could be construed as a potential conflict of interest.
